# Raised Soluble P-Selectin Moderately Accelerates Atherosclerotic Plaque Progression

**DOI:** 10.1371/journal.pone.0097422

**Published:** 2014-05-20

**Authors:** Kevin J. Woollard, Natalie G. Lumsden, Karen L. Andrews, Andrea Aprico, Emma Harris, Jennifer C. Irvine, Ann-maree Jefferis, Lu Fang, Peter Kanellakis, Alex Bobik, Jaye P. F. Chin-Dusting

**Affiliations:** Baker IDI Heart and Diabetes Institute, Melbourne, Victoria, Australia; Thomas Jefferson University, United States of America

## Abstract

Soluble P-selectin (sP-selectin), a biomarker of inflammatory related pathologies including cardiovascular and peripheral vascular diseases, also has pro-atherosclerotic effects including the ability to increase leukocyte recruitment and modulate thrombotic responses *in vivo*. The current study explores its role in progressing atherosclerotic plaque disease. *Apoe* ^−/−^ mice placed on a high fat diet (HFD) were given daily injections of recombinant dimeric murine P-selectin (22.5 µg/kg/day) for 8 or 16 weeks. Saline or sE-selectin injections were used as negative controls. In order to assess the role of sP-selectin on atherothrombosis an experimental plaque remodelling murine model, with *sm22α-hDTR Apoe^−/−^* mice on a HFD in conjunction with delivery of diphtheria toxin to induce targeted vascular smooth muscle apoptosis, was used. These mice were similarly given daily injections of sP-selectin for 8 or 16 weeks. While plaque mass and aortic lipid content did not change with sP-selectin treatment in *Apoe^−/−^* or *SM22α-hDTR Apoe^−/−^* mice on HFD, increased plasma MCP-1 and a higher plaque CD45 content in *Apoe* ^−/−^ HFD mice was observed. As well, a significant shift towards a more unstable plaque phenotype in the *SM22α-hDTR Apoe^−/−^* HFD mice, with increased macrophage accumulation and lower collagen content, leading to a lower plaque stability index, was observed. These results demonstrate that chronically raised sP-selectin favours progression of an unstable atherosclerotic plaque phenotype.

## Introduction

Cardiovascular disease (CVD) remains the largest cause of death in the world [1]. A vital mechanism for the development of an unstable atherosclerotic plaque is through increased vascular leukocyte recruitment, which is part of the inflammation hypothesis of atherosclerosis in CVD [2]. Critically, inflammatory plaques lead to extracellular matrix remodelling and vascular smooth muscle cell (VSMC) destruction [2]. These characteristics have been utilised experimentally by generating a VSMC inducible knockout mouse, the *SM22α-hDTR Apoe^−/−^*_,_ that presents with a vulnerable plaque phenotype and evidence of plaque rupture [3].

P-selectin is a member of the selectin family, is localised in the membranes of the α-granules of platelets and the Weibel-Palade bodies of endothelial cells and is expressed on the surface of activated platelets and endothelial cells [4]. It contributes to leukocyte recruitment at sites of vascular injury and inflammation and acts via the engagement of the ligand PSGL-1 [5]–[7]. Previous studies have shown that platelet P-selectin engagement of PSGL-1 leads to Mac-1 activation and subsequent firm adhesion [8], [9]. P-selectin deficiency or antibody-mediated inhibition of its adhesive function reduces early atherogenesis in animal models [10], [11]. The soluble form of P-selectin (sP-selectin), arising from either proteolytic cleavage or secretion of an alternatively spliced isoform, is increased in disease states prompting its identification as a potentially valuable clinical biomarker of vascular disease risk [12], [13].

We have previously demonstrated that the elevated levels of sP-selectin present in patients with peripheral vascular disease is not only a biomarker of disease but has functional effects by activating leukocytes and promoting their adhesion to platelet monolayers [14]. We have also shown that sP-selectin promotes leukocyte adhesion through PSGL-1 outside-in signalling through Src and PI3K, leading to Mac-1 integrin activation and subsequent recruitment to arterial and venule micro- and macro-circulation [15], a finding described by others [16].

Given that raised sP-selectin appears to be mediating increased arterial leukocyte recruitment, and more recent data showing that raised sP-selectin can directly affect atherothrombosis in a transgenic mouse model (deltaCT/deltaCT) [17], we hypothesised that chronically raised sP-selectin will exacerbate disease progression in genetically susceptible mouse models of atherosclerosis.

## Materials and Methods

### Materials

Recombinant murine dimeric soluble E- and P-selectin-Fc Chimera were purchased from R & D systems (USA). Both recombinant proteins were endotoxin free as analysed by the manufacturer and confirmed in independent limulus assays, giving values below 0.01 EU/ml. Diptheria toxin was from *Corynebacterium diphtheriae* and purchased from Sigma-Aldrich (USA).

### Animals

All procedures and protocols were approved by the AMREP Animal Ethics Committee (project approval number: E/0972/2010/B) and conformed to the Guide for the Care and Use of Laboratory Animals (NIH).

Male *Apoe^−/−^* mice on the *C57BL/6* background and male *SM22α-hDTR Apoe^−/−^* were bred and housed at the Precinct Animal Centre, Alfred Medical Research and Education Precinct (AMREP), Melbourne, Australia. *SM22α-hDTR Apoe^−/−^* mice were created based on the published model described by Clarke *et al* [3]. Briefly, the mouse SM22α promoter (−445 to +88 relative to transcriptional start site) was cloned into the pSTEC-1/2 vector upstream of the mDTR-eGFP sequence. DNA was prepared for injection of the vector into oocytes and implantation and generation of chimeric progenitors was provided by the Biomolecular Institute (Australia). *SM22a-hDTR* mice were born at the expected frequency and developed normally. *SM22a-hDTR* were subsequently crossed with *ApoE^−/−^* and progeny developed normally with no difference in phenotype from *ApoE^−/−^*. From 6 weeks of age mice received daily sub-cutaneous (s.c) injections of either vehicle (saline), sE-selectin (22.5 µg/kg/day) or sP-selectin (22.5 µg/kg/day) and were fed high fat diet (HFD) containing 0.15% cholesterol and 22% fat (Speciality Feeds, Western Australia) for 8 or 16 weeks. The dose of selectins was chosen on the basis of raising plasma sP-selectin by approximately 150 ng/ml, which is observed in human pathologies, most notably in cardiovascular and peripheral vascular disease, as previously described [15]. *SM22α-hDTR Apoe^−/−^* mice and their *Apoe^−/−^* mice controls were injected with Diptheria toxin (5 ng/g; three times weekly) for the last three weeks of the HFD.

### Histology

The proximal aorta from within the heart was dissected and sectioned into sinus root, arch, thoracic and abdominal areas and embedded in OCT compound and snap frozen with isopentane chilled by liquid nitrogen. 6–10 µm sections of each aortic segment were analysed for lesion size defined as the cross sectional surface area of Oil Red O staining within the aortic intima, or by immunohistochemistry to identify CD45 (Pharminogen), CD68 (Serotec) and alpha smooth muscle actin (αSMA) expression (Abcam). Briefly, sections were fixed in cold (−20°C) acetone for 20 min. The sections were then sequentially incubated in 3% hydrogen peroxide in PBS, 10% normal goat/horse serum/PBS and biotin/avidin blocking reagents (Vector Laboratories). Thereafter, the sections were incubated with primary antibodies in normal goat serum. Sections were incubated with the corresponding biotynylated secondary antibodies and detected using the Avidin Biotin staining Complex (Vector Laboratories) with DAB (3,3′-Diaminobenzidine) reagent (Vector Laboratories). DAB treated sections were counterstained with hematoxylin and Scotts tap water. Apoptotic cells were identified by the *in situ* cell death detection kit dUTP nick end -labelling with peroxidase label (TUNEL-POD) (Roche). Collagen was stained with 0.1% Sirius Red F3BA (Sigma-Aldrich) in isopropanol and lipid with Oil Red O or Sudan-IV. Necrotic core was determined by analysis of Mayer’s Hematoxylin and Eosin stained sections with the unstained cellular regions defined as necrotic, as previously described [18].

Images were taken using an FSX100 Olympus microscope and analysed using Fiji 1.47 h software. Stained areas were expressed as a percentage of total plaque area. Stained cells (eg. TUNEL and CD45) were expressed as a percentage of total plaque cells. All mean data including mean lesion size was calculated from the measurement of cross sections taken from every 60 µm of the first 180 µm of the aortic sinus root proximal to the aortic arch or from every 100 µm of the first 600 µm in the ascending aorta proximal to the heart. Only advanced plaques (defined as those displaying necrotic cores) were analysed in this study. Plaque stability scores were defined as ratio of collagen content over lipid area, as previously described [19].

### Sudan En Face Staining

Aortae segments (abdominal, thoracic, arch) were cleaned of peri-aortic fat, stained with Sudan IV and imaged by a light microscope (Motic SMZ-168) connected to a digital camera and computer. Stained areas were quantified using Fiji 1.47 h software and expressed as a percentage of total aorta area.

### Plasma Protein/Cytokine/Chemokine Measurements

Blood was collected via cardiac puncture and plasma stored in sodium citrate (129 mM) tubes and analysed for soluble E- and P-selectin levels using ELISA (R & D) and mouse MCP-1 (CCL2), Rantes (CCL5), TNF-α, MIP-1α (CCL3), and IL-1β content using a Bio-Plex assay kit (Bio-Rad CA, U.S.A), according to the manufacturers’ instructions.

### Statistical Analysis

Results are expressed as mean ± SEM. Comparisons between groups were carried out using a student’s unpaired t-test or for multiple comparisons, by a one way ANOVA followed by Tukey’s post-hoc test. A value of *P*<0.05 was considered as statistically significant. At least 6 mice per group were analysed for each data set.

## Results

### Effect of Treatment on Plasma Soluble Markers in Apoe^−/−^ Mice

**Figure 1:**
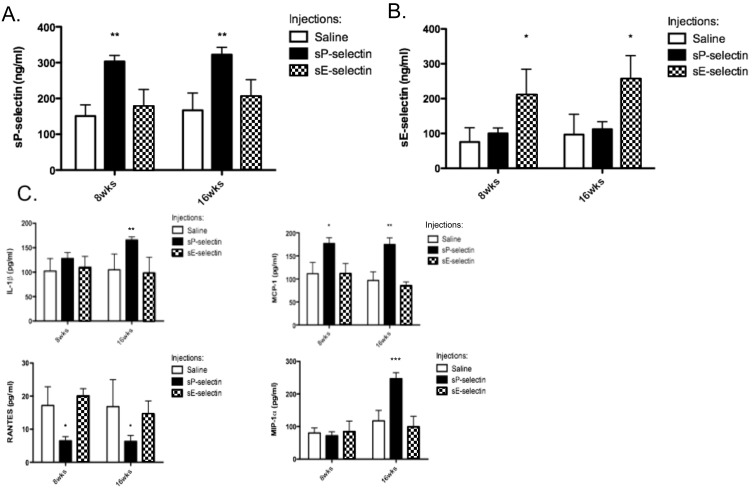
sP-selectin, sE-selectin and cytokine/chemokine plasma levels in *ApoE^−/−^* HFD mice receiving daily injections of recombinant sP-selectin or sE-selectin. **A)** Plasma levels of sP-selectin after 8 or 16 weeks of daily s.c injections (22.5 µg/kg/day) of vehicle control (saline matched volume; white bar), sP-selectin (black bar) or sE-selectin (checkered bar). **B)** Plasma levels of sE-selectin after 8 or 16 weeks of daily s.c injections (22.5 µg/kg/day) of vehicle control (saline matched volume; white bar), sP-selectin (black bar) or sE-selectin (checkered bar). **C)** Plasma levels of IL-1β, MCP-1, MIP-1α and RANTES after 8 or 16 weeks of daily s.c injections (22.5 µg/kg/day) of vehicle control (saline matched volume; white bar), sP-selectin (black bar) or sE-selectin (checkered bar). *n* = 9–15 mice per treatment group. Data represented as Mean (ng or pg/ml) ± SEM where *, ** and ** represents *P*<0.05, <0.01 and <0.001 respectively (from saline treatment) as analysed by One-way ANOVA with Tukey’s post-hoc test.

Plasma sP-selectin and sE-selectin levels were analysed in all animals. Figure 1A shows that following either 8 or 16 weeks of daily injections of recombinant sP-selectin, circulating plasma levels increased to around 300 ng/ml, which is significantly higher than levels observed in saline or sE-selectin treated animals, and is comparable to human pathologies, including CVD and PVD [13], [20]. Similarly sE-selectin injections led to increases in plasma levels of sE-selectin as measured by ELISA (figure 1B).

To generate a picture of the overall systemic inflammatory response following treatments, a plasma BioPlex ELISA was performed. sP-selectin treatment significantly increased plasma levels of IL-1β, MCP-1 and MIP-1α (figure 1C), while a significant decrease in RANTES was also noted (figure 1C).

### Effect of sP-selectin on Apoe^−/−^ Plaque Size and Phenotype

**Figure 2:**
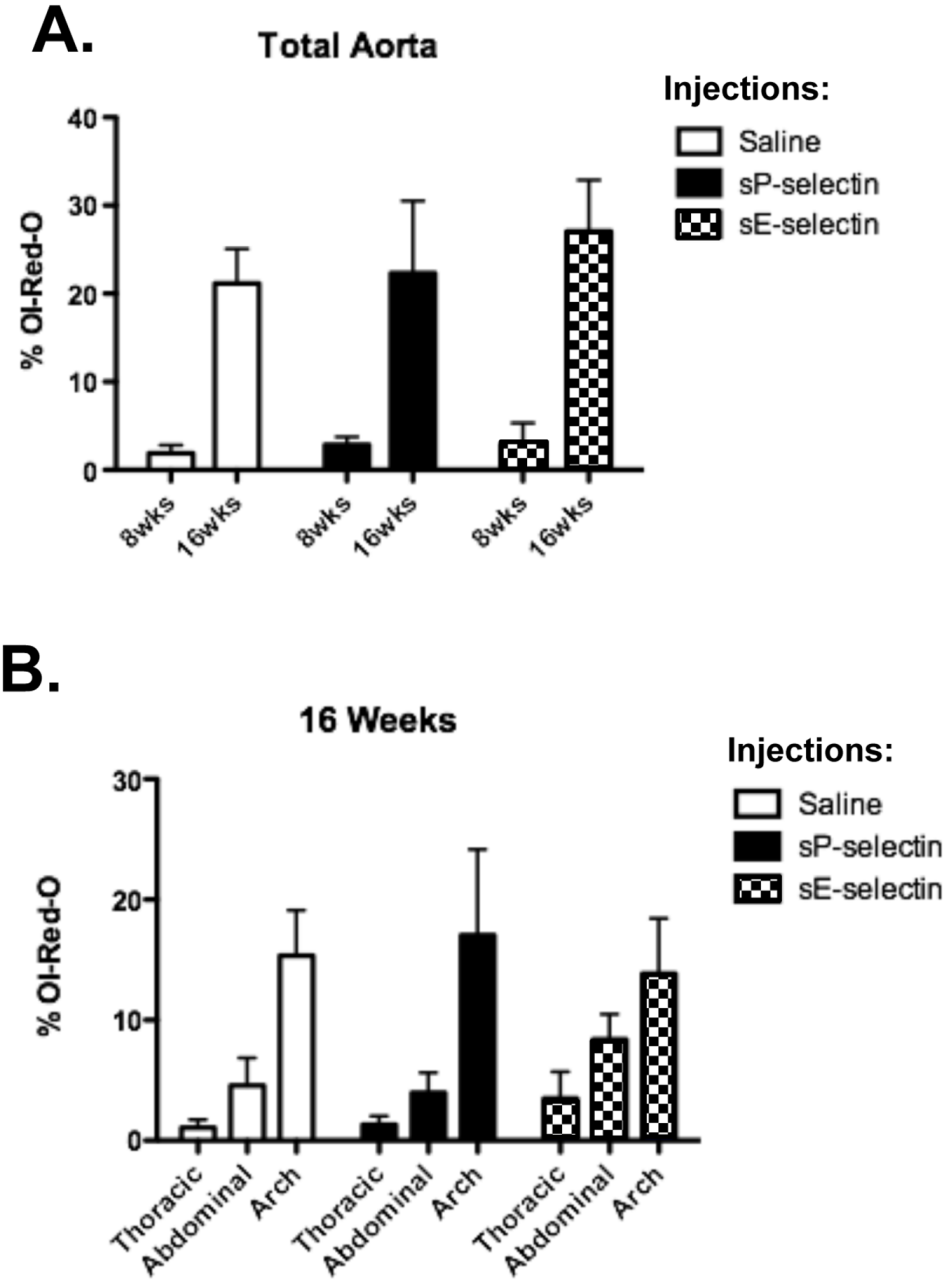
Oil-Red-O assessment of plaque area in *ApoE^−/−^* on HFD with sP-selectin treatment. Sections of each aortic segment were analysed for lesion size defined as the cross sectional surface area of Oil Red O staining within the aortic intima **A)** from total aorta after 8 or 16 weeks of daily s.c injections (22.5 µg/kg/day) of vehicle control (saline matched volume; white bar), sP-selectin (black bar) or sE-selectin (checkered bar), **B)** from indicated aortic sections after 16 weeks of daily s.c injections (22.5 µg/kg/day) of vehicle control (saline matched volume; white bar), sP-selectin (black bar) or sE-selectin (checkered bar). *n* = 6–9 mice per treatment group. Data represented as Mean (% ORO) ± SEM.

Figure 2 shows plaque size assessed as percentage of Oil-red-O staining in a given field, from daily injections of sP-selectin, or sE-selectin and saline delivered as controls, into HFD fed *Apoe* ^−/−^ mice fed. As expected, aortic sections had significantly greater atherosclerosis in all groups of *Apoe* ^−/−^ mice from 8 to 16 weeks on HFD (figure 2a). However, there were no significant differences between treatment groups. Plaque mass was also assessed for thoracic, abdominal and arch areas. While arch areas did show significantly increased atherosclerosis compared to either the thoracic or abdominal regions, there were no changes in atherosclerosis between treatment groups at either 8 weeks (data not shown) or 16 weeks of treatment (figure 2b).

**Figure 3:**
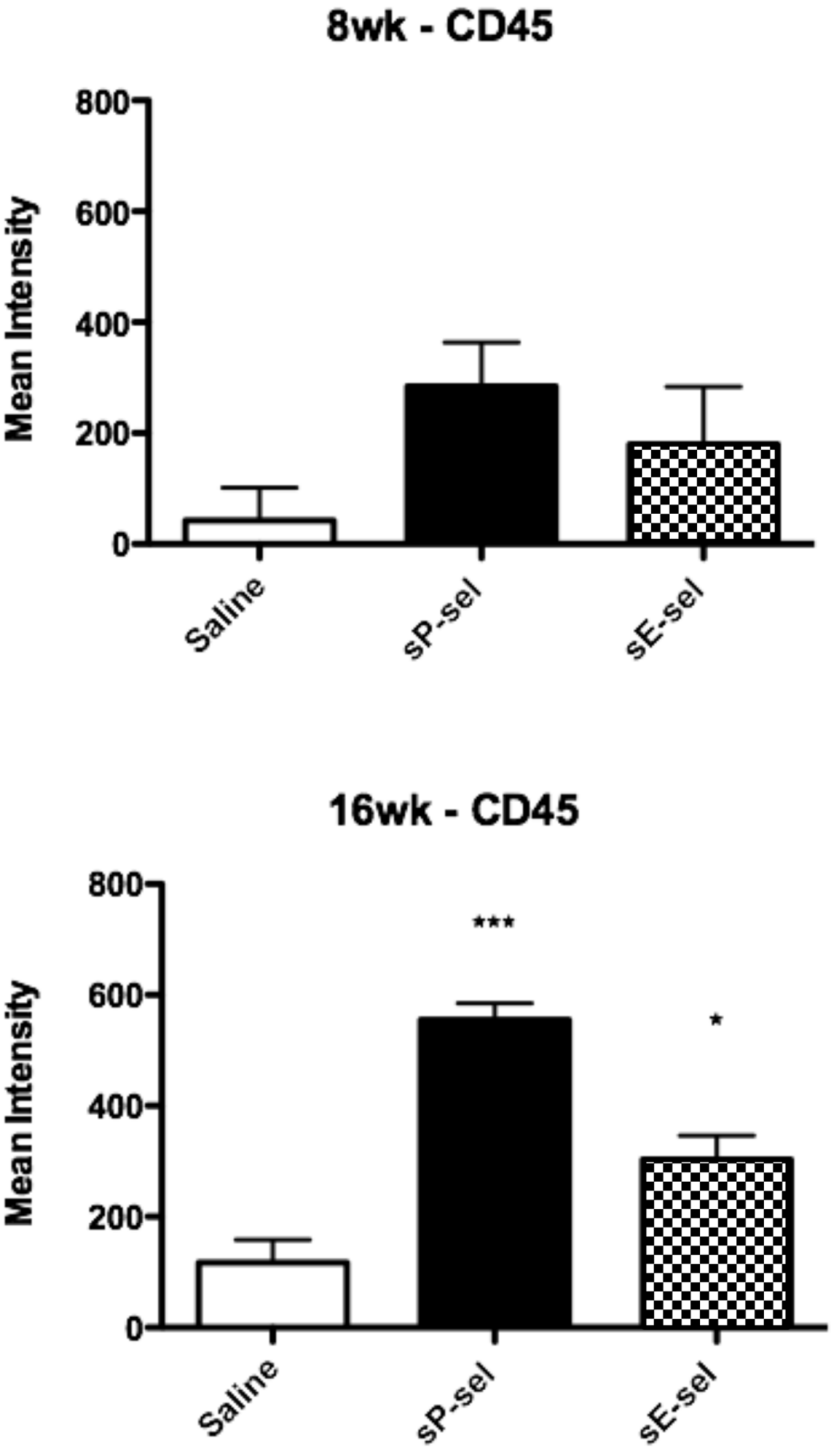
Aortic CD45^+^ expression in *ApoE^−/−^* HFD after sP-selectin treatment. Sections of each aortic segment were analysed for CD45^+^ positive cells by immunohistochemistry after 8 or 16 weeks of daily s.c injections (22.5 µg/kg/day) of vehicle control (saline matched volume; white bar), sP-selectin (black bar) or sE-selectin (checkered bar). *n* = 6–9 mice per treatment group. Data represented as Mean (DAB intensity) ± SEM where * and *** represents *P*<0.05 and <0.001 respectively (from saline treatment) as analysed by One-way ANOVA with Tukey’s post-hoc test.

Figure 3 shows a trend for an increase in plaque CD45^+^ leukocytes after 8 weeks of sP-selectin treatment which became highly significant at 16 weeks. A small but significant increase in plaque CD45^+^ leukocyte content following 16 weeks of sE-selectin administration was also observed, although this was half of that observed following sP-selectin (figure 3).

### Effect of sP-selectin on Plasma Inflammatory Markers in SM22α-hDTR Apoe^−/−^

To further explore the effect of s-P-selectin on plaque phenotype, we applied a published experimental plaque remodelling model (*SM22α-hDTR Apoe^−/−^*) [3] in combination with raising plasma sP-selectin over 8 and 16 wks.

**Figure 4:**
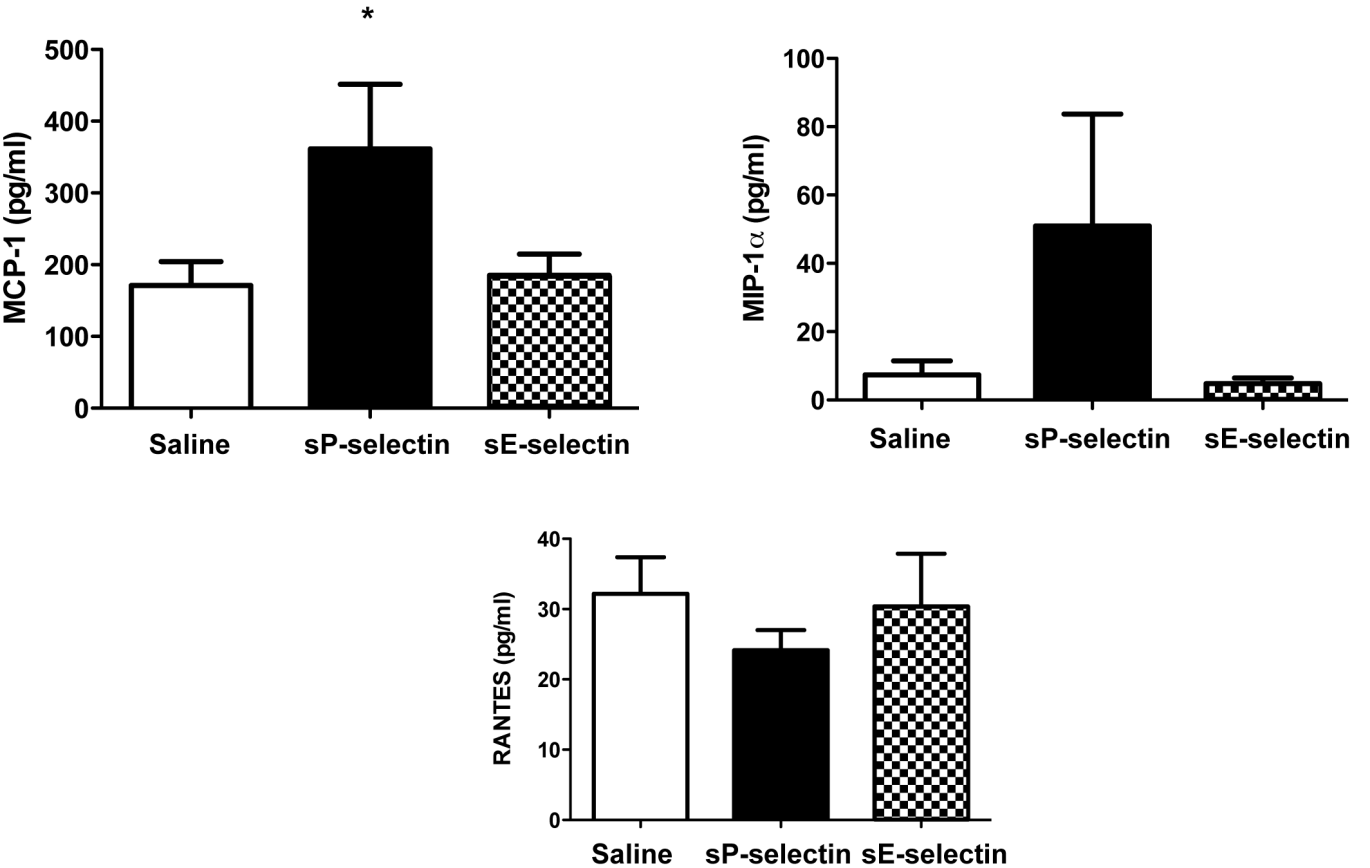
Plasma cytokine and chemokine levels after sP-selectin treatment for 16weeks in *SM22α-hDTR ApoE^−/−^* HFD. Plasma levels of MCP-1, MIP-1α and RANTES after 8 or 16 weeks of daily s.c injections (22.5 µg/kg/day) of vehicle control (saline matched volume; white bar), sP-selectin (black bar) or sE-selectin (checkered bar). *n* = 9–15 mice per treatment group. Data represented as Mean (pg/ml) ± SEM where * represents *P*<0.05 (from saline treatment) as analysed by unpaired students t-test.

To examine if similar changes in plasma inflammatory markers could also be noted in the *SM22α-hDTR Apoe^−/−^* model as in *Apoe^−/−^* alone (on HFD), we analysed plasma levels of MCP-1, MIP-1α, IL1β and RANTES after sP-selectin or control treatments (saline, sE-selectin) after 16 wks (8 wk data not shown). Similar to *Apoe^−/−^* on HFD alone, sP-selectin increased plasma levels of MCP-1 and MIP-1α in this model (figure 4). This was coupled with a decrease in RANTES after sP-selectin treatment (figure 4). There was no difference in IL1β levels (data not shown).

### Effect of sP-selectin on Plaque Morphology in SM22α-hDTR Apoe^−/−^

**Figure 5:**
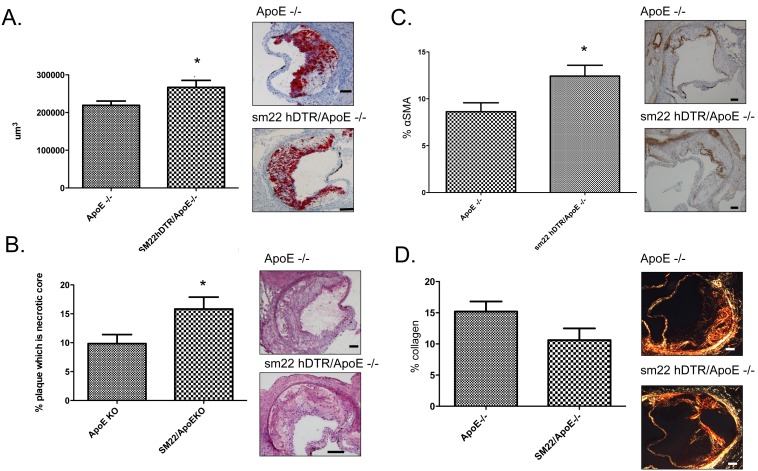
Model validation data. Sections of each aortic segment were analysed for lesion size, necrotic core, αSMA and collagen content in *Apoe* ^−/−^ HFD or *SM22hDTR-ApoE^−/−^* HFD mice, **A)** lesion size defined as % ORO staining, **B)** necrotic core defined as % unstained with Mayer’s Hematoxylin and Eosin, **C)** % α-SMA content, **D)** % collagen content. *n* = 6–9 mice per treatment group. Data represented as Mean (% stain) ± SEM where * represents *P*<0.05 as analysed by unpaired students t-test. Representative images are given for each analysis. Scale bars = 100 µm.

A preliminary validation group of 16 wk HFD fed *SM22α-hDTR Apoe^−/−^* in comparison to *Apoe^−/−^* mice showed a significant change in overall plaque mass (figure 5A). There was a significant increase in necrotic core and αSMA content with a decrease in collagen content (figure 5B–D). These data confirm previous findings using the *SM22α-hDTR Apoe^−/−^* mouse model, albeit with less significance [3].

**Figure 6:**
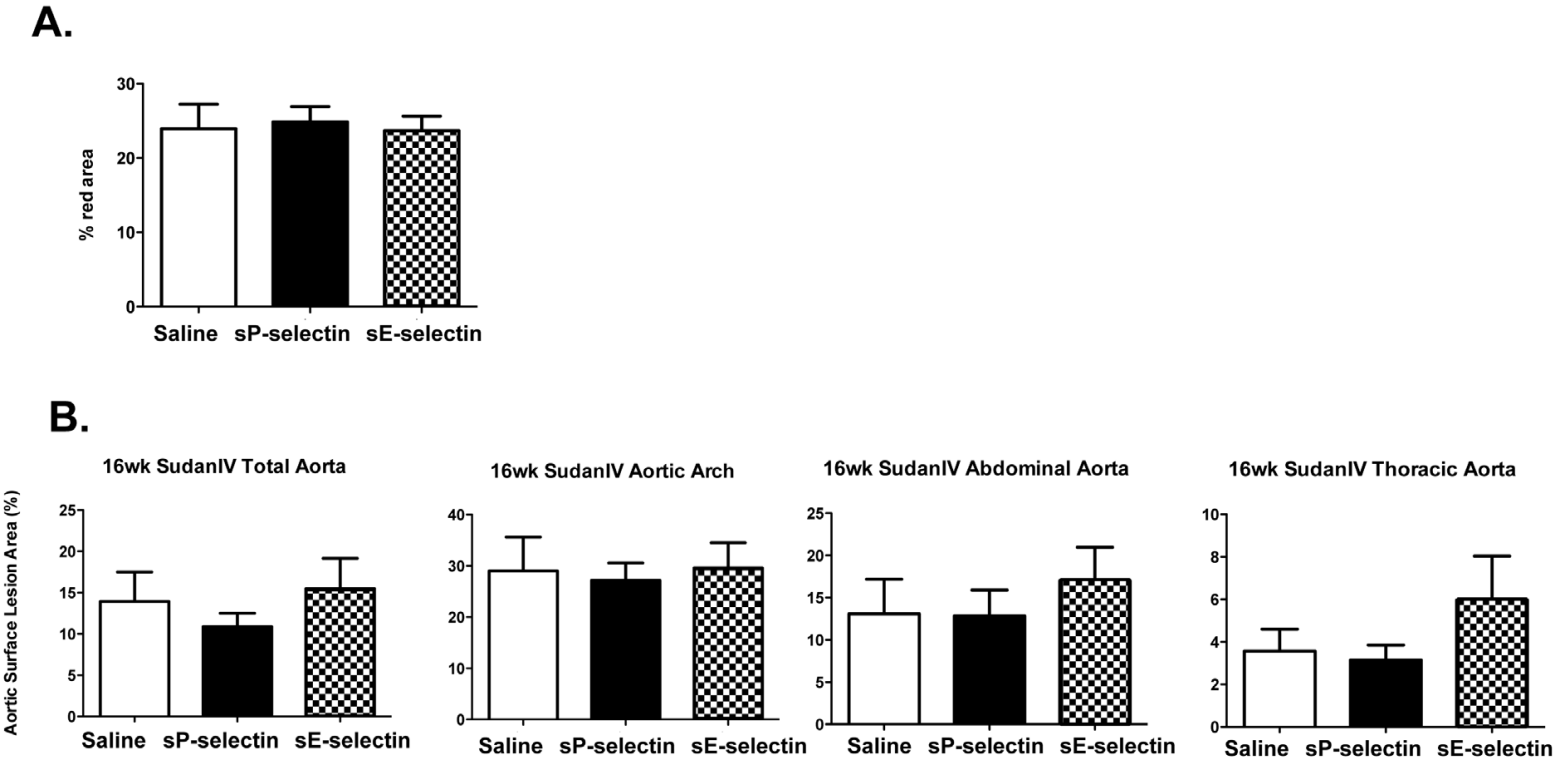
Oil-Red-O and *en face* Sudan IV assessment of plaque area in *SM22α-hDTR ApoE^−/−^* HFD with sP-selectin treatment for 16 weeks. **A)** Sections of each aortic segment were analysed for lesion size defined as the cross sectional surface area of % ORO staining within the aortic intima after 16 weeks of daily s.c injections (22.5 µg/kg/day) of vehicle control (saline matched volume; white bar), sP-selectin (black bar) or sE-selectin (checkered bar). **B)** En-face Sudan IV staining from indicated aortic sections after 16 weeks of daily s.c injections (22.5 µg/kg/day) of vehicle control (saline matched volume; white bar), sP-selectin (black bar) or sE-selectin (checkered bar). *n* = 6–9 mice per treatment group. Data represented as Mean (%)±SEM.

We next applied daily injections of sP-selectin, sE-selectin or saline for 8 and 16 weeks. Figure 6 shows that, as in *Apoe* ^−/−^ mice, *SM22α-hDTR Apoe^−/−^* mice on a HFD with daily injections of sP-selectin for 16 weeks exhibited no significant change in total plaque mass lipid content (ORO), as compared to control injections using saline or sE-selectin. This was also noted in all regions of the aorta using *en-face* Sudan IV staining after 16 wk treatment (figure 6B). No change in lipid content was noted after 8 wk treatments (data not shown).

**Figure 7:**
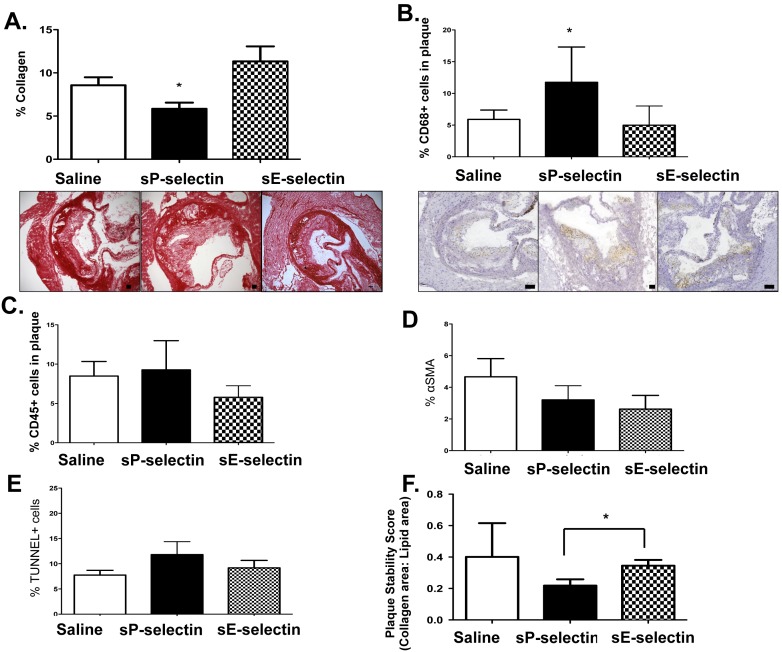
Collagen, CD45^+^, CD68^+^, α-SMA and apoptotic cell content and plaque stability scores in *SM22α-hDTR ApoE^−/−^* HFD after sP-selectin treatment for 16 weeks. **A)** Sections of each aortic segment were analysed for collagen content by immunohistochemistry after 16 weeks of daily s.c injections (22.5 µg/kg/day) of vehicle control (saline matched volume; white bar), sP-selectin (black bar) or sE-selectin (checkered bar). Representative images are given below. Sections of each aortic segment were analysed for **B)** CD68^+^ (representative images shown below) and **C)** CD45^+^ cells by immunohistochemistry after 16 weeks of daily s.c injections (22.5 µg/kg/day) of vehicle control (saline matched volume; white bar), sP-selectin (black bar) or sE-selectin (checkered bar) **D)** αSMA content or **E)** apoptotic cell content (% TUNEL) by immunohistochemistry after 16 weeks of daily s.c injections (22.5 µg/kg/day) of vehicle control (saline matched volume; white bar), sP-selectin (black bar) or sE-selectin (checkered bar). **F)** Plaque stability scores were defined as ratio of collagen content over lipid area after 16 weeks of daily s.c injections (22.5 µg/kg/day) of vehicle control (saline matched volume; white bar), sP-selectin (black bar) or sE-selectin (checkered bar). *n* = 6–9 mice per treatment group. Data represented as Mean (% stain or score) ± SEM where * represents *P*<0.05 (from saline or indicated treatment) as analysed by One-way ANOVA with Tukey’s post-hoc test or unpaired student’s t-test (C, D). Scale bars = 100 µm.

Conversely, sP-selectin treatment led to significantly lower collagen content after 16 weeks (figure 7A) and a significantly higher CD68^+^ macrophage accumulation (figure 7B) but not total CD45^+^ cells in the plaque (figure 7C), compared to saline and E-selectin injected controls (8 wk data not shown). No change in collagen content was noted after 8 wk treatments (data not shown) and there was no effect of treatment on αSMA content and apoptotic cells (figure 7D–E) at 16 weeks. Overall a significant reduction in plaque stability score, as assessed by collagen over plaque area, was observed (figure 7F).

## Discussion

Like all selectins, membrane bound P-selectin has an N-terminal lectin domain, an epidermal growth factor motif, and specifically, nine regulatory protein repeats, a transmembrane section and a short intracytoplasmic tail [6]. After activation of endothelial cells by inflammatory mediators such as TNFα, Weibel–Palade bodies can become rapidly mobilised, which results in P-selectin expression extending approximately 40 nm from the endothelial surface, lasting up to 3 hours depending on the vasculature [6], [21]. Similarly, platelets can undergo surface expression of P-selectin upon activation by agonists such as P2 receptor agonists [22]. As P-selectin is a component of the membrane of platelet alpha and dense granules, expression reflects activation [22], [23]. P-selectin is a potent adhesion molecule and has been reported to have procoagulant activities by regulating the production of monocyte derived platelet activating factor and tissue factor [24]. Moreover, P-selectin primes monocytes for increased phagocytosis [25]. However, the role of P-selectin in vascular inflammation is complicated by a circulating soluble form, which can arise from proteolytic cleavage and direct expression of P-selectin lacking the cytoplasmic domain [26]. Evidence that sP-selectin has a direct role in atherosclerosis has been reported [17]. Studies from our laboratory demonstrate a role for sP-selectin in regulating leukocyte adhesion in patients with peripheral arterial occlusive disease *in-vivo* [14], [15]. Pathophysiological concentrations of sP-selectin engages its ligand, PSGL-1, resulting in Src kinase-dependent Mac-1 (CD11b/CD18) up-regulation and adhesive function [14], [15]. Overall, these earlier studies raised the possibility that sP-selectin may promote leukocyte recruitment to sites of vessel wall injury and vascular endothelium in patients with CVD.

We wanted to explore this issue and examine if chronically raised plasma levels of sP-selectin led to direct changes in atherosclerosis progression and/or phenotype in the *Apoe* ^−/−^ experimental mouse model on HFD. Daily injections of 22.5 µg/kg/day of recombinant Fc-chimera sP-selectin over 8 and 16 weeks, led to significantly raised plasma levels of protein which were similar to pathophysiological levels seen in CVD [13], [20]. We showed that chronically raising plasma levels of sP-selectin had modest effects on atherosclerosis in *Apoe* ^−/−^ mice on HFD, compared to raised sE-selectin and saline injection controls. When initially examining total plaque mass, we observed no significant change in atherosclerosis over 8 and 16 weeks HFD with sP-selectin, compared to controls. This was disappointing given previous reports using the DeltaCT mouse model which exhibits abnormally high concentrations of plasma sP-selectin [27], leading to increased aortic sinus lesion mass after 16 wks on normal chow [17]. These disparate findings may represent variations in effects due to the structure of sP-selectin, which in the DeltaCT model lacks the cytoplasmic domain [27]. Indeed, the DeltaCT mouse model demonstrates significant increases in procoagulant activity [27], which may account for its indirect pro-atherosclerotic effects. Also plasma levels of sP-selectin were approximately 3 fold higher than described here and in clinical human disease [20]. Nevertheless, there was marked plaque infiltration of CD45^+^ leukocytes after sP-selectin treatment for 16 weeks with *Apoe* ^−/−^ on HFD, with notable increases in plasma inflammatory markers, including the monocyte specific chemokine MCP-1. Therefore, we speculated that chronically raising plasma levels of sP-selectin may alter plaque stability.

In order to test the hypothesis that raised plasma sP-selectin increases plaque vulnerability we employed a plaque destabilising model using the genetically susceptible mouse model, *SM22α-hDTR* on *Apoe* ^−/−^ and HFD background [3]. Previous work has investigated VSMC apoptosis by generating transgenic mice that express the human diphtheria toxin receptor (hDTR), encoded by heparin-binding EGF-like growth factor from a minimal Tagln (also known as SM22α) promoter [3]. In this model, 50–70% of VSMC apoptosis resulted in normal arteries with no inflammation, thrombosis, remodeling or aneurysm formation [3]. However, the atherosclerotic plaques with VSMC apoptosis in *SM22α-hDTR Apoe* ^−/−^ mice resulted in thinning of the fibrous cap, loss of collagen and intimal inflammation, which are all features of plaque vulnerability [3], [28].

VSMCs together with extracellular matrix components comprise the medial layer of adult arteries. VSMC apoptosis can increase as atherosclerotic plaques develop and rupture [29] [28]. After this initial wave of apoptosis, normal medial cell content can be derived several weeks later [30], suggesting that VSMC apoptosis may initiate repopulation. Moreover, VSMC apoptosis may induce calcification, coagulation and be pro-inflammatory [31]. Importantly, VSMC apoptosis causes release of IL-1α [32], MCP-1 and IL-8 resulting in infiltration of macrophages [33], as we find in sP-selectin treated *SM22α-hDTR Apoe* ^−/−^ mice.

Overall, we would speculate that VSMC apoptosis may lead to evidence of unstable plaque or silent plaque rupture in mouse models of atherosclerosis and that raising plasma sP-selectin levels, would increase the risk of plaque rupture, as suggested by others in the DeltaCT model [17]. As such, we performed a preliminary validation group of 16 wk HFD fed *SM22α-hDTR Apoe^−/−^* in comparison to *ApoE^−/−^* mice on HFD. The *SM22α-hDTR Apoe^−/−^* mice showed significant changes in necrotic core, collagen content, increased apoptosis and αSMA content, but was absent of changes in overall plaque mass. These data confirm previous findings of Clarke *et al* [3], albeit with reduced significance, and demonstrate that *SM22α-hDTR Apoe^−/−^* HFD background mice exhibit signs of unstable plaque, leading to an overall decreased plaque stability score, as is noted in human disease [34]. Interestingly, raising plasma sP-selectin in *SM22α-hDTR Apoe^−/−^* HFD mice led to decreased atherosclerotic collagen content and increased CD68^+^ macrophage plaque infiltration, compared to controls. Moreover, similar to sP-selectin treatment in *Apoe^−/−^* HFD alone, sP-selectin treatment in *SM22α-hDTR Apoe^−/−^* HFD mice elicited increased plasma levels of MCP-1 and MIP-1α.

MCP-1 and MIP-1α are important chemokines involved in monocyte recruitment, which is integral to vascular inflammation and atherosclerosis. The MCP-1 and MIP-1α chemokine axis may be important in regulating recruitment of specific subsets of monocytes with independent effector functions, specifically CCR2^high^CD14^high^CD16^l^°^w^ and CX3CR1^high^CD14^l^°^w^CD16^high^ human monocytes [35]–[37]. The contribution of these monocyte subsets in atherosclerosis is part of ongoing work by many labs, in which recruitment of CCR2^high^CD14^high^ monocytes is thought to be particularly important in inflammatory atherosclerosis [35], [38]. More work will be needed to understand if soluble P-selectin mediates differences in monocyte subset numbers or recruitment, and macrophage phenotype in atherosclerosis. Further, in light of a recent study showing that inflammatory plaque progression may be regulated by tissue macrophage proliferation independent of monocyte recruitment [39], the role of monocyte recruitment in vulnerable atherosclerosis progression requires further investigation. Neovascularisation has been noted as a hallmark of atherogenesis and a mechanism by which monocytes can enter atherosclerotic lesions [40]. We did not analyse neovascularisation in our model as it was beyond the scope of the current study. Future studies are necessary, however, to investigate whether unstable plaque progression with high levels of sP-selectin promotes neovascularisation and macrophage accumulation.

RANTES is expressed by T-cells, fibroblasts, mesangial cells and platelets [41], [42] and when overexpressed in atherosclerosis, can engage chemokine receptors on the endothelium, mediating transmigration of monocytes and lymphocytes into the intima [43], [44]. RANTES is also expressed in atherosclerotic plaques [45]. Given this strong association between inflammatory atherosclerosis and RANTES, we would have expected an increase in plasma levels of RANTES after sP-selectin. Unexpectedly, we noted significant decreases in RANTES in our *Apoe^−/−^* HFD model. Of interest, recent work in the Atherosclerosis Risk in Communities (ARIC) Carotid MRI study has shown that mean minimum fibrous cap thickness was positively associated with RANTES levels [46]. As the thickness of the fibrous cap dictates the stability of the atherosclerotic plaque [47], it may be that RANTES levels compensate for increased inflammatory burden after sP-selectin treatment. This hypothesis requires further investigation.

Overall, asides from significant decreases in collagen content and significant increases in plaque inflammatory cell infiltrates and plasma inflammatory chemokines, raising plasma levels of sP-selectin in *SM22α-hDTR ApoE^−/−^* on HFD as a model of experimental unstable atherosclerosis only led to modest changes in plaque stability scores, as compared to controls. However, collagen content is a primary characteristic of unstable/vulnerable plaque and strategies that have directly targeted collagen content through either experimental regression studies or therapeutically increasing plaque collagen density have proven to have positive outcomes in experimental models of atherosclerosis [48]. Therefore this reduction in collagen with sP-selectin treatment, coupled with macrophage infiltration and systemic inflammatory chemokines, would lead to a significant increased risk of plaque rupture and warrants further investigation as to whether targeting raised plasma sP-selectin would be an effective therapeutic strategy in unstable CVD.

In conclusion, we have shown in two models of experimental atherosclerosis that raising plasma concentrations of sP-selectin to pathophysiological levels had some effects on plaque phenotype related to characteristics of vulnerable plaque, such as reduced collagen content and increased inflammatory cellular content. We propose that an increase in circulating sP-selectin may not only be a marker of vascular disease, but may also contribute to the inflammatory hypothesis of unstable plaque progression.
